# Exploring salivary metabolome alterations in people with HIV: towards early diagnostic markers

**DOI:** 10.3389/fpubh.2024.1400332

**Published:** 2024-06-05

**Authors:** Fei Du, Rong Li, Rui He, Kezeng Li, Jun Liu, Yingying Xiang, Kaiwen Duan, Chengwen Li

**Affiliations:** ^1^Department of Stomatology, Yan’an Hospital of Kunming City, Yan’an Hospital Affiliated to Kunming Medical University, Kunming, Yunnan, China; ^2^Department of Stomatology, The First Affiliated Hospital of Dali University, Dali, Yunnan, China; ^3^Department of Stomatology, Kunming Maternal and Child Health Hospital, Kunming, Yunnan, China; ^4^Department of Infectious Diseases, Kunming Third People’s Hospital, Kunming, Yunnan, China; ^5^Department of Research Management, Third Affiliated Hospital of Kunming Medical University, Kunming, Yunnan, China

**Keywords:** HIV, salivary metabolomics, highly active antiretroviral therapy (HAART), LC–MS/MS, biomarker identification

## Abstract

**Background:**

The human immunodeficiency virus (HIV) remains a critical global health issue, with a pressing need for effective diagnostic and monitoring tools.

**Methodology:**

This study explored distinctions in salivary metabolome among healthy individuals, individuals with HIV, and those receiving highly active antiretroviral therapy (HAART). Utilizing LC–MS/MS for exhaustive metabolomics profiling, we analyzed 90 oral saliva samples from individuals with HIV, categorized by CD4 count levels in the peripheral blood.

**Results:**

Orthogonal partial least squares-discriminant analysis (OPLS-DA) and other analyses underscored significant metabolic alterations in individuals with HIV, especially in energy metabolism pathways. Notably, post-HAART metabolic profiles indicated a substantial presence of exogenous metabolites and changes in amino acid pathways like arginine, proline, and lysine degradation. Key metabolites such as citric acid, L-glutamic acid, and L-histidine were identified as potential indicators of disease progression or recovery. Differential metabolite selection and functional enrichment analysis, combined with receiver operating characteristic (ROC) and random forest analyses, pinpointed potential biomarkers for different stages of HIV infection. Additionally, our research examined the interplay between oral metabolites and microorganisms such as herpes simplex virus type 1 (HSV1), bacteria, and fungi in individuals with HIV, revealing crucial interactions.

**Conclusion:**

This investigation seeks to contribute understanding into the metabolic shifts occurring in HIV infection and following the initiation of HAART, while tentatively proposing novel avenues for diagnostic and treatment monitoring through salivary metabolomics.

## 1 Introduction

The infection of human immunodeficiency virus (HIV) leads to acquired immunodeficiency syndrome (AIDS), marking one of the most significant pandemics in history (1). HIV spreads through blood, bodily fluids, and organ and tissue transplantation (2–4). Typically, new HIV transmissions predominantly occur in individuals who are unaware of their health status (5). HIV invasion causes cell-mediated immune deficiency, primarily due to the depletion of circulating CD4+ T cells (6, 7). As a retrovirus, HIV enters the body and binds to macrophages and dendritic cells, which then transport the virus to CD4+ T cells. Infected CD4+ T cells home to lymphatic tissues, where the virus replicates and spreads extensively, establishing a persistent infection. Immune activation, a critical driver of HIV replication, is mediated by abnormal cellular signaling due to the secretion of various cytokines and the interaction of the viral envelope with cellular receptors. Ultimately, this results in lymphocyte depletion and the disruption of lymphatic tissue structures, leading to immunological damage (7). Owing to compromised immunity, the control of wound infections in people living with HIV (PLWH) becomes challenging, leading to complications in wound healing and infection, which can trigger comorbidities (8). HIV infection increases the risk of numerous comorbidities and opportunistic infections, including cardiac disease (9), uveitis (10), and head and neck squamous cell carcinoma (HNSCC) (11), thereby increasing mortality rates.

Current research on HIV primarily focuses on HIV subtyping (12), disease and treatment dynamics, regional infection trends and improvement strategies (13), as well as prevention and care (14). Studies on metabolic profiles in PLWH have concentrated on oral disease-related metabolites (4), neural metabolites (15), plasma metabolites (16), and fungal metabolites (17). It has been found that HIV RNA, proviral DNA, and infected cells are readily detectable in the salivary secretions of individuals with HIV, with saliva collection being a painless, simple, and rapid method (1). Saliva contains molecular information related to HIV, and its easy collection suggests its potential as a substitute for blood measurements. Studies have shown that the salivary metabolomic profile of individuals with HIV differs from that of individuals without HIVs (4). However, little is known about the salivary metabolomics in PLWH. Thus, there is an urgent need for salivary biomarkers associated with HIV infection. The use of untargeted metabolomics could facilitate the identification of these biomarkers and further elucidating their mechanisms of action.

Highly active antiretroviral therapy (HAART) is widely used in HIV treatment. It has been proven to be effective in suppressing systemic HIV-1 viral load, reducing mortality rates, and lowering the incidence of opportunistic infections in PLWH (18, 19). In this study, we aimed to explore the differences in salivary metabolites among normal controls, individuals with HIV, and individuals with HIV receiving HAART. A total of 90 saliva samples from PLWH were collected, with patients grouped according to peripheral blood CD4 count levels. Subsequent saliva samples were collected after the introduction of HAART for LC–MS/MS metabolomic sequencing. This method allows for the untargeted analysis of saliva samples before and after HAART. It facilitates the detection of salivary metabolic changes, with the goal of identifying biomarkers related to HAART. Through these analyses, we hope to provide healthcare professionals with valuable information for diagnosing and assessing HIV conditions by examining changes in oral metabolites in PLWH.

## 2 Materials and methods

### 2.1 Sample collection and grouping

This study amassed oral saliva samples from 90 individuals with HIV, each providing one sample, by the Department of Stomatology of Yan’an Hospital, Kunming. Patients were categorized into the HIV group based on peripheral blood CD4 counts and subdivided into CD4-L (CD4 count <200/mm3), CD4-M (CD4 count 200-500/mm3), and CD4-H (CD4 count >500/mm3), with each subgroup comprising 30 individuals. The control group (CON) consisted of healthy individuals matched in age, gender, ethnicity, and dietary habits with the case group, all being generally healthy and voluntary participants. Exclusion criteria included: pregnant or lactating women, individuals with impaired consciousness, dementia, psychiatric disorders, diabetes, severe oral lesions, recent use of antibiotics, and those unable to tolerate oral examination. Follow-up oral fluid samples were collected 6, 12, and 18 months after highly active anti-retroviral therapy (HAART), labeling them as the THIV group, denoted as T-6, T-12, and T-18, respectively. Demographic data of participants are demonstrated in Table 1.

**Table 1:** Characteristics of the study population.

		HARRT duration				Non-medication
		6 months	12 months	18 months	*p*-valve	
Male (%)		51%	58%	53%	0.7791^a^	69%
Age		43 ± 12	36 ± 13	37 ± 12	0.0173^b^	38 ± 13
HIV staging	I	30	27	29		58
	II, III, IV	10	13	16		10
Possible route of infection	Drug addiction	5	4	8		4
	Sexual behavior	35	39	37		80
						
Before HARRT	CD4 count	4 ~ 727	8 ~ 698	7 ~ 618	0.442^b^	13.9 ~ 973
	CD8 count	140 ~ 4,212	293 ~ 2,352	74 ~ 2,246	0.239^b^	35 ~ 3,373
	Viral load	100 ~ 9,999,999	649 ~ 3,717,000	1,463 ~ 1,323,000	0.225^b^	110 ~ 330,000
						
After HARRT	CD4 count	31.9 ~ 956.6	56.5 ~ 2253.6	109.5 ~ 1,066	0.769^b^	-
	CD8 count	179 ~ 2510.9	286 ~ 1992	77.6 ~ 1717.8	0.154^b^	-
	Viral load	-	-	-	-	-

a *p* value is based on chi-square test.

b *p* values are based on ANOVA test.

A written informed consent was acquired from all participants before inclusion. The study was approved by the Medical Ethics Committee of Yan’an Hospital in Kunming, Yunnan Province.

### 2.2 Metabolite extraction

Initially, 200 μL of each sample was transferred into a 1.5 mL Eppendorf tube, to which 350 μL of extraction liquid (V methanol: V acetonitrile: V H2O = 2:2:1) was added, along with 20 μL L-2-Chlorophenylalanine (1 mg/mL in dH2O) as an internal standard. Samples were vortex mixed for 30 s, followed by ultrasound treatment for 10 min in ice water. After incubating at −20°C for 1 h, samples were centrifuged at 13800 g for 15 min at 4°C. The supernatant (0.5 mL) was then transferred to a fresh 1.5 mL Eppendorf tube and dried in a vacuum concentrator without heating. The obtained dry extract was reconstituted with 300 μL of extraction liquid (V acetonitrile: V water = 1:1), vortexed for 30 s, and sonicated in a 4°C water bath for 10 min. Following another centrifugation at 12000 rpm for 15 min at 4°C, 60 μL of the supernatant was transferred into a fresh 2 mL LC/MS glass vial, with 10 μL from each sample pooled as QC samples. Afterwards, 60 μL of supernatant was then used for UHPLC-QTOF-MS analysis. Data collection was segmented by mass range (50–300, 290–600, 590–900, 890–1,200) to expand the collection rate of the secondary spectra. Four replicates were collected for each method segment.

### 2.3 Data processing

Data processing was conducted using the xcms4dda and xcms4lipid programs based on XCMS, setting minfrac to 0.5 and cutoff to 0.1. Secondary data were initially screened, retaining peaks identified in either forward or reverse analysis. Peaks from primary and secondary data were then matched primarily based on mz and RT, within an mz tolerance at ±30 ppm and RT tolerance at ±60s. Prior to data analysis, data tables were normalized using the SVR algorithm. QC samples with detection rates <50% and RSD > 30% were omitted, leading to the final data compilation.

### 2.4 Orthogonal partial least squares-discriminant analysis (OPLS-DA)

After the acquisition of metabolomic sequencing data, OPLS-DA was applied to observe intergroup sample differences. Model accuracy and reliability were evaluated using the respective orthogonal T scores. Further visualization of significant metabolites in the HIV and THIV groups was achieved through analysis of variance (ANOVA), heatmap, and hierarchical clustering analyses, conducted using R packages ropls (v. 1.30.0) and pheatmap (v. 1.0.12), respectively.

### 2.5 Differential metabolite selection and functional enrichment analysis

Significant differential metabolites were selected based on the variable importance in projection (VIP) from the OPLS-DA model, with VIP > 1 and *p* < 0.05 indicating significance. Differential metabolites were annotated using the Kyoto Encyclopedia of Genes and Genomes (KEGG) compound database,^1^ and subsequently mapped to the KEGG pathway database. Pathways with significant modulation of metabolites were illustrated, with their significance assessed via hypergeometric test *p*-values. Key KEGG enriched pathways were then selected, with metabolite accumulations displayed in heatmaps and metabolite pathways depicted using AI tools.

### 2.6 Trend analysis

Metabolites in both the HIV and THIV groups underwent time trend analysis using the TCseq R package (v. 1.22.6). Clusters with similar trends were chosen for KEGG enrichment analysis. Finally, the levels of metabolites within enriched pathways were visualized in heatmaps.

### 2.7 Metabolite correlation analysis

All metabolites, including those in weighted correlation network analysis (WGCNA) modules, were subjected to Pearson correlation analysis, with correlation coefficients and corresponding heatmaps generated using R (v. 4.2.3). The relationships among autocorrelated metabolites were visualized using Cytoscape (v. 3.10.0), with certain network results visualized using the MCODE (v. 2.0.3) plugin.

### 2.8 Weighted correlation network analysis

The WGCNA package (v. 1.72–5) in R was employed to scrutinize metabolites within the metabolome. Metabolites from significant modules were analyzed using analysis of ANOVA, with their distribution exhibited in heatmaps. Subsequently, metabolites in each module underwent network analysis to observe inter-metabolite interactions. WGCNA and network interactions were analyzed using R package and Cytoscape.

### 2.9 Identification of biomarkers

Receiver operating characteristic curve (ROC) analysis and the random forest algorithm were performed on detected metabolites to identify biomarkers. ROC curves were generated via Monte-Carlo cross-validation (MCCV) using balanced sub-sampling. In each MCCV, 2/3 of the samples were used to evaluate feature importance, and the top 5, 10, 15, 20 important features were then utilized to build classification models, which were validated on the 1/3 of the samples left out. This procedure was repeated multiple times to calculate the performance and confidence interval of each model. The random forest classification method was then employed for metabolite ranking. Finally, feature metabolites were selected based on their areas under the curve (AUCs) for validation, resulting in ROC curves.

### 2.10 Taqman PCR analysis of HSV-1, bacteria and fungi in saliva

Absolute quantification of HSV-1, as well as bacteria and fungi related to oral diseases was performed using Taqman PCR. Specifically, these bacteria primarily included *Staphylococcus aureus*, *Escherichia coli*, *Pseudomonas aeruginosa*, *Proteus mirabilis*, *Streptococcus pneumoniae*, *Klebsiella pneumoniae*, and others. The fungi included *Candida albicans*, *Candida albicans*, *Candida tropicalis*, *Aspergillus niger*, and *Aspergillus flavus*. The Gg1(US4) gene of HSV-1, 16S ribosomal RNA (rRNA) gene for bacteria, and 18S rRNA gene for fungi were used as targets for detection and quantification, following the methods described in a previous report (20). Primer sequences of HSV-1 were designed using Primer Premier 5.0, while those of bacteria and fungi were retrieved from SILVA SSU r114 database accessed through the SILVA website.^2^ Primers were synthesized by Invitrogen (CA, United States), with sequences demonstrated in Table 2.

**Table 2:** Primer sequences of HSV-1, bacteria, and fungi.

Type	Detection Loci	Primers and Probes (5′-3′)
HSV-1 (Herpes simplex virus type 1)	Gg1(US4)	HHV-1 F: CTGTTCTCGTTCCTCACTGCCT
		HHV-1 R: CAAAAACGATAAGGTGTGGATGAC
		HHV-1 OUT_F: TCGAGAAGGACAAACCCAAC
		HHV-1 OUT_R: CGCACCAATACACAAAAACG
		HHV-1 P: 5-(FAM)CCCTGGACACCCTCTTCGTCGTCAG(TAMRA)-3
Bacteria	*16S* rRNA gene	F: GCAACGCGAAGAACCTTACC
		R: ACGTCATCCCCACCTTCCT
		Probe:FAM-ACGACAACCATGCACCACCTG-TAMRA
Fungi	18 s rRNA gene	F:CTGGCGATGGTTCATTCAAA
		R:CTTGCCCTCCAATTGTTCCT
		Probe:FAM-TAAGGGTTCGATTCCGGAG-TAMRA

## 3 Results

### 3.1 Comprehensive metabolomics profiling revealed distinct alterations in PLWH

**Figure 1:**
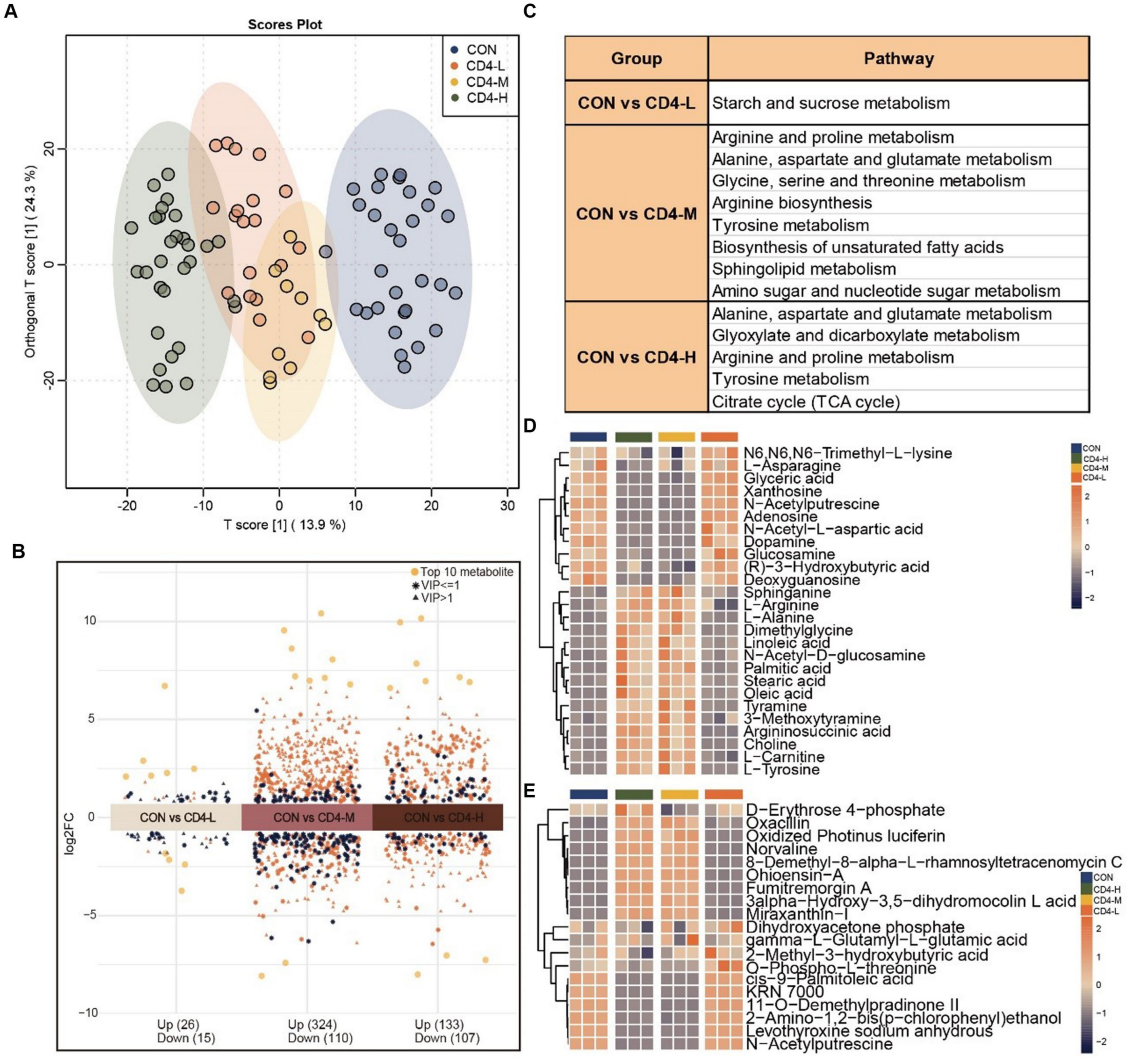
Metabolomic analysis of pre-HAART HIV group. **(A)** OPLS-DA score plot differentiating the HIV group (CD4-L, CD4-M, CD4-H) from the normal control group (CON). **(B)** Differential metabolites between the three pre-HAART HIV groups and the control group, considered significant with VIP > 1, Log2FC > 0, *p* < 0.05. **(C)** KEGG enrichment analysis of the differential metabolites in the pre-HAART HIV groups (*p* < 0.05). **(D)** Accumulation profiles of metabolites in enriched KEGG pathways, with orange indicating high levels and blue indicating low levels. **(E)** Analysis of variance (ANOVA) highlighting the 19 most significant metabolites, with high level in orange and low level in blue (*p* < 0.05).

By integrating metabolites from both positive and negative ion modes in LC–MS/MS analysis, a total of 1,401 mixed data points were obtained. To delineate the metabolic alterations between pre- and post-HAART individuals with HIV more distinctly, differential, functional, and trend analyses were conducted. It was revealed that the metabolites in PLWH were significantly enriched in energy metabolism. The construction of an OPLS-DA model aimed to identify outliers and cluster patterns within the overall sample set. Utilizing the T-score, partial overlap among the CD4-L, CD4-M, and CD4-H groups was observed. However, a distinct separation from the CON group was observed, indicating a high degree of sample differentiation between the HIV and CON groups (Figure 1A). In the analysis of differential metabolites between the CON and HIV groups, 41 (upregulated 26, downregulated 15), 434 (upregulated 324, downregulated 110), and 240 (upregulated 133, downregulated 107) significantly varied metabolites were identified in the CD4-L, CD4-M, and CD4-H groups, respectively (Figure 1B). Further KEGG functional enrichment analysis revealed that the CD4-L group was enriched in starch and sucrose metabolism. The CD4-M group showed enrichment in arginine and proline metabolism, glycine, serine and threonine metabolism, biosynthesis of unsaturated fatty acids, sphingolipid metabolism, and amino sugar and nucleotide sugar metabolism. In addition, both CD4-M and CD4-H were enriched in tyrosine metabolism, alanine, aspartate, and glutamate metabolism, and arginine and proline metabolism (Figure 1C). An examination of metabolite levels within these pathways revealed a reverse relationship when comparing the CON and CD4-L groups with the CD4-M and CD4-H groups. Metabolites such as L-asparagine, glyceric acid, xanthosine, adenosine, dopamine, glucosamine, and deoxyguanosine had lower levels in CD4-M and CD4-H, whereas sphinganine, L-arginine, L-alanine, and dimethylglycine were more abundant (Figure 1D). ANOVA revealed similar metabolite levels between the CON and CD4-L groups, as well as between the CD4-M and CD4-H groups. Metabolites such as 3alpha-hydroxy-3,5-dihydromocolin L acid and miraxanthin-I were more prevalent in CD4-M and CD4-H, whereas the accumulation of cis-9-palmitoleic acid, KRN 7000, 11-O-demethylpradinone II, 2-amino-1,2-bis (p-chlorophenyl) ethanol, and N-acetylputrescine was inversely related (Figure 1E).

**Figure 2:**
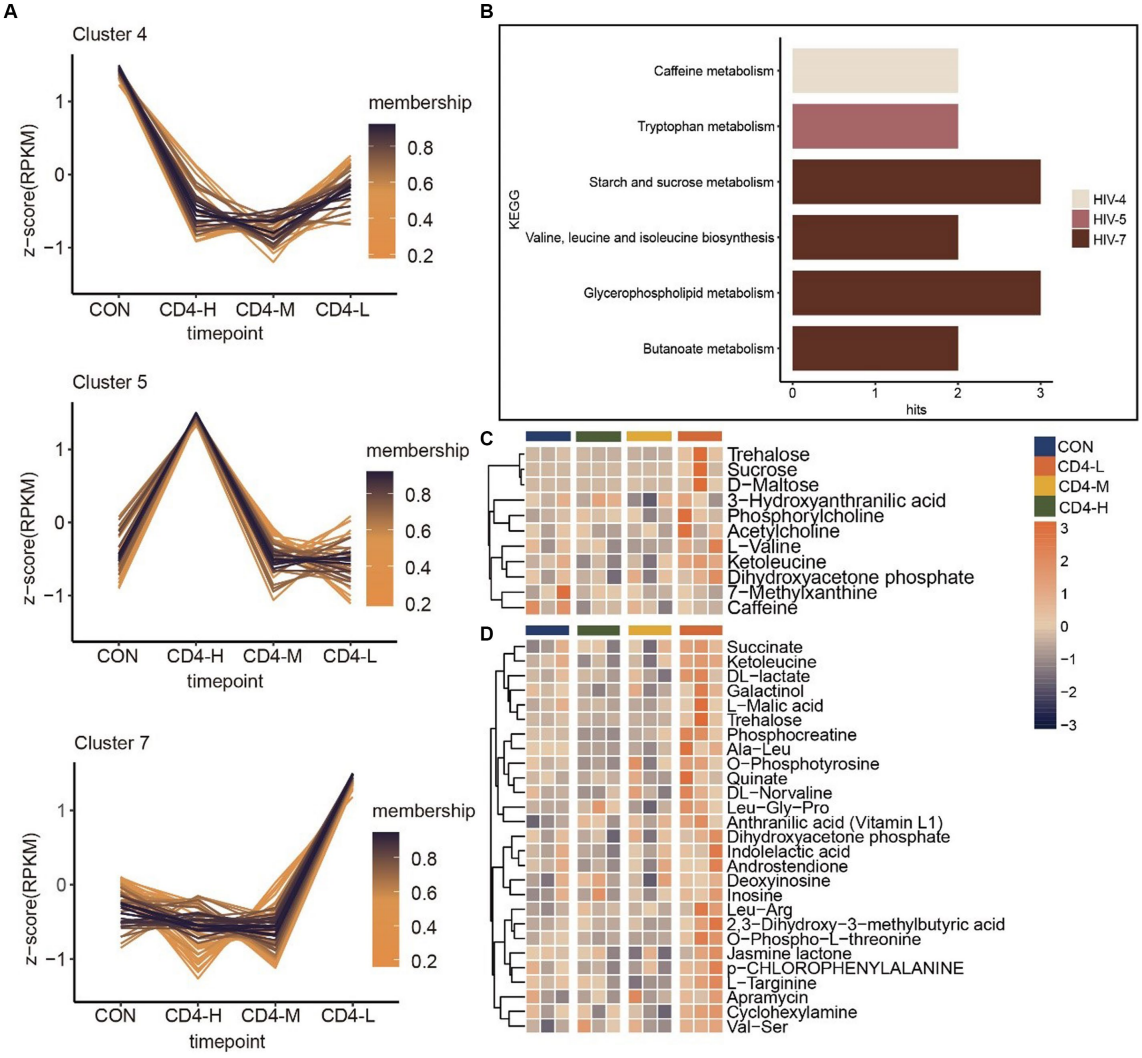
Trend analysis of metabolites in pre-HAART PLWH as well as associated metabolic pathways and metabolite accumulation. **(A)** Trend analysis of metabolites in pre-HAART PLWH. **(B)** KEGG enrichment in three clusters (*p* < 0.05). **(C)** Levels of metabolites in pathways, with high levels in orange and low levels in blue. **(D)** Metabolic levels of metabolites in Cluster 7.

Next, by trend analysis and functional analysis for all metabolites, we identified distinct patterns in Cluster 4, Cluster 5 and Cluster 7. Cluster 4 metabolites demonstrated a decreasing trend with diminishing CD4 cell counts, albeit a slight increase when the count was below 200. KEGG enrichment analysis linked these metabolites to caffeine metabolism. Cluster 5 metabolites initially rose after HIV infection, but they showed a decreasing trend as the disease progressed, with enrichment in tryptophan metabolism. In contrast, Cluster 7 metabolites progressively increased with the advancement of HIV infection, implicating butanoate metabolism, glycerophospholipid metabolism, valine, leucine and isoleucine biosynthesis, and starch and sucrose metabolism (Figures 2A,B). Further analysis of the metabolic content in pathways revealed that metabolites in the CD4-L group were highly expressed compared to the other three groups, especially trehalose and ketoleucin. Conversely, L-valine and 3-hydroxyanthranilic acid exhibited lower levels in the CD4-M group (Figure 2C). Lastly, given the alignment of Cluster 7 metabolic trends with the progression of HIV infection, the metabolic profile of this Cluster was analyzed, revealing a significant increase in the CD4-L group (Figure 2D).

### 3.2 Severity of PLWH after HAART linked to distinct metabolomic profiles in THIV group

**Figure 3:**
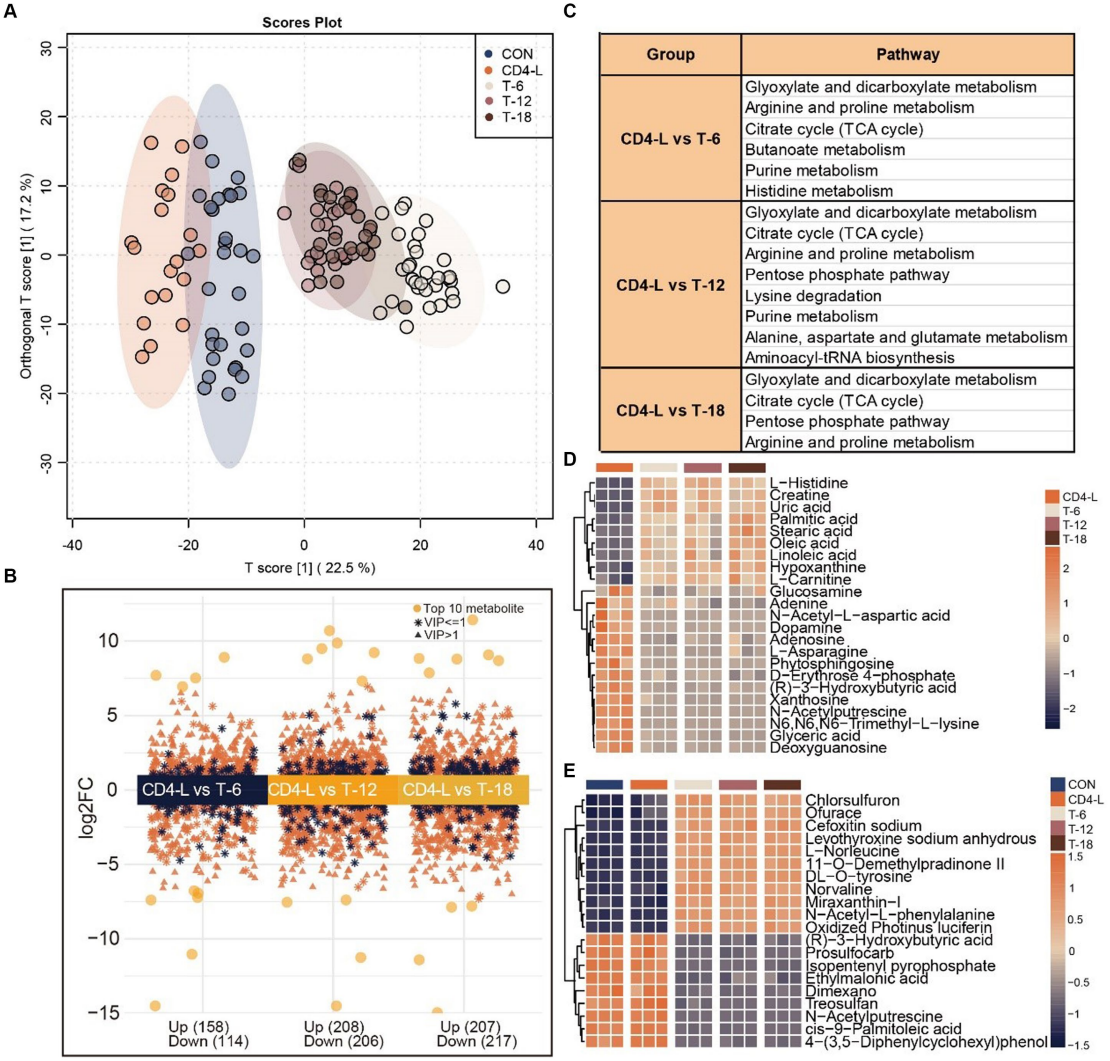
Metabolomic analysis of the THIV group. **(A)** OPLS-DA score plot showed clear separation of the THIV group from the control and CD4-L groups. **(B)** Differential metabolites between the three stages of the THIV group (T-6, T-12, T-18) and the CD4-L group, with significance determined by VIP > 1, Log2FC > 0, and *p* < 0.05. **(C)** KEGG enrichment analysis of the differential metabolites in the three stages of the THIV group (*p* < 0.05). **(D)** Metabolite profile in the KEGG enriched pathways, with high level in orange and low level in blue. **(E)** ANOVA showing the 20 most significant metabolites, with high levels in orange and low levels in blue (*p* < 0.05).

To identify metabolites significantly associated with the severity of PLWH after the introduction of HAART, a comprehensive metabolomic analysis of the THIV group was conducted. This revealed a predominance of exogenous metabolites. OPLS-DA distinctly separated the THIV group from the CD-4 group, with the T-6, T-12, and T-18 samples clustering together and well-separated from the CON and CD4-L groups, demonstrating a clear delineation between treated and untreated samples (Figure 3A). This distinction was also reflected in the metabolite content analysis. A differential metabolite analysis yielded 272 (upregulated 158, downregulated 114), 414 (upregulated 208, downregulated 206), and 424 (upregulated 207, downregulated 217) significantly varied metabolites in the T-6, T-12 and T-18 groups, respectively (Figure 3B). Enrichment analysis indicated that glyoxylate and dicarboxylate metabolism, arginine and proline metabolism, and the citrate cycle (TCA cycle) were commonly enriched across the three groups. T-6 exhibited enrichment in butanoate metabolism and histidine metabolism. T-12 showed enrichment in lysine degradation, alanine, aspartate, and glutamate metabolism, and aminoacyl-tRNA biosynthesis. T-18 was enriched in arginine and proline metabolism (Figure 3C). Subsequent quantification of metabolites within these pathways revealed an inverse relationship between the metabolite content and of the treated group and the CD4-L group. Notable increases were observed in the levels of L-histidine, linoleic acid, hypoxanthine, and L-carnitine after treatment. Conversely, glucosamine, adenine, N-acetyl-L-aspartic acid, and L-asparagine had higher levels in the CD4-L group than in the THIV group (Figure 3D). ANOVA revealed that exogenous metabolites such as L-norleucine, DL-O-tyrosine, norvaline, and N-acetyl-L-phenylalanine were elevated after treatment, while (R)-3-hydroxybutyric acid and N-acetylputrescine showed inverse patterns (Figure 3E).

**Figure 4:**
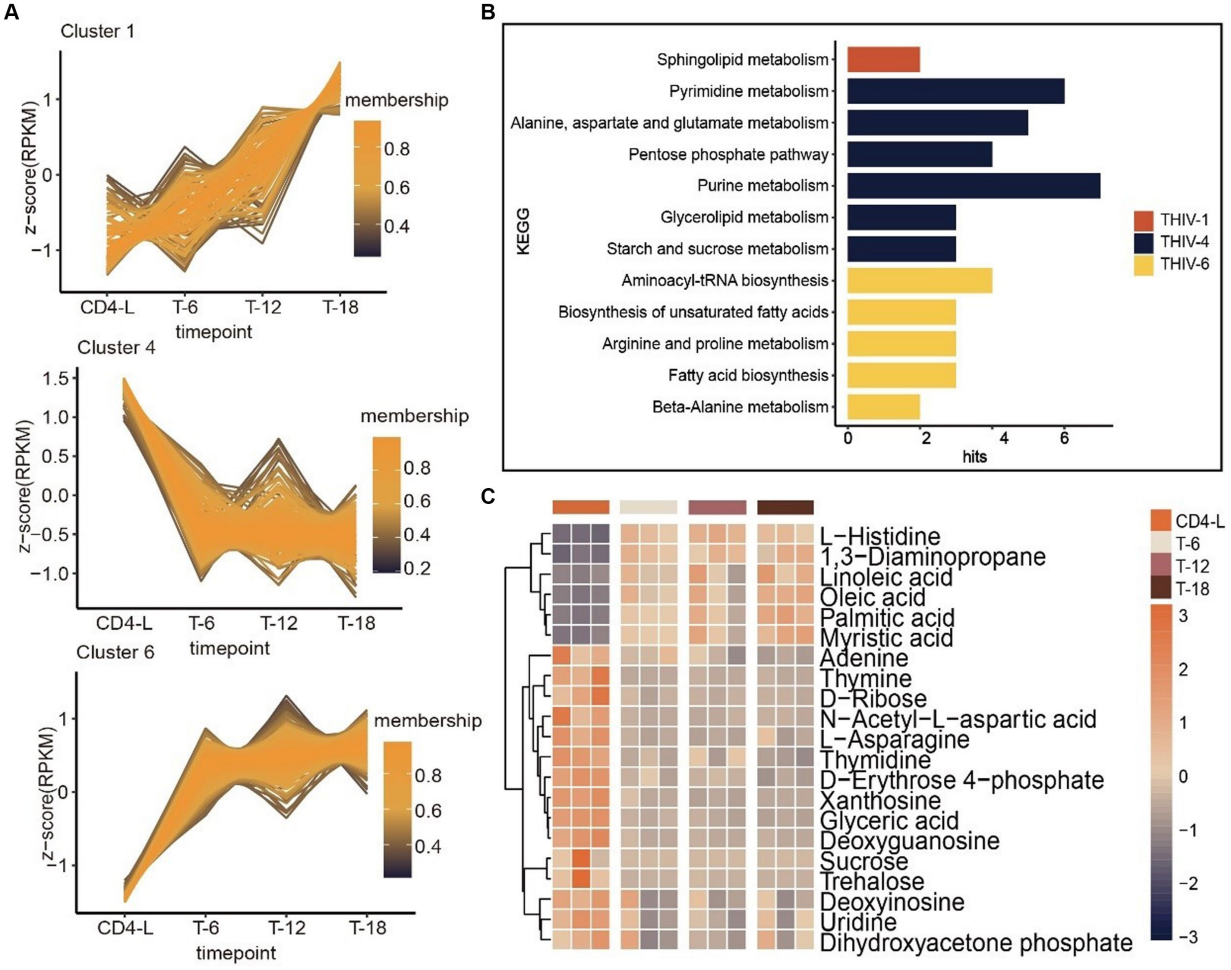
Trend analysis of PLWH after HAART, as well as associated metabolic pathways and metabolite accumulation. **(A)** Trend analysis of metabolites in PLWH after treatment revealed three clusters with characteristic trajectories. **(B)** KEGG enrichment of metabolites in the three clusters. **(C)** Accumulation levels of metabolites in the pathways, with high levels in orange and low levels in blue.

Further trend analysis of metabolites throughout the treatment stages identified characteristic trajectories in Cluster 1, 4 and 6. The metabolites in Cluster 1 exhibited a consistent upward trend throughout the course of treatment, enriched in sphingolipid metabolism. Metabolites in Cluster 4 demonstrated a prompt declining trend upon treatment, but experienced less pronounced changes over time. This cluster was enriched in starch and sucrose metabolism, glycerolipid metabolism, purine metabolism, the pentose phosphate pathway, alanine, aspartate and glutamate metabolism, and pyrimidine metabolism. In contrast, the trajectory of Cluster 6 was opposite to Cluster 4, with metabolites enriched in beta-alanine metabolism, fatty acid biosynthesis, arginine and proline metabolism, biosynthesis of unsaturated fatty acids, and aminoacyl-tRNA biosynthesis (Figures 4A,B). A quantitative review of pathway-associated metabolites showed an inverse accumulation compared with the CD4-L group. Metabolites such as L-histidine, 1,3-diaminopropane, linoleic acid, and oleic acid increased after treatment. Conversely, adenine, L-asparagine, D-erythrose 4-phosphate, xanthosine, glyceric acid, and deoxyguanosine decreased (Figure 4C).

### 3.3 HIV infection altered amino acid and energy metabolism in saliva

**Figure 5:**
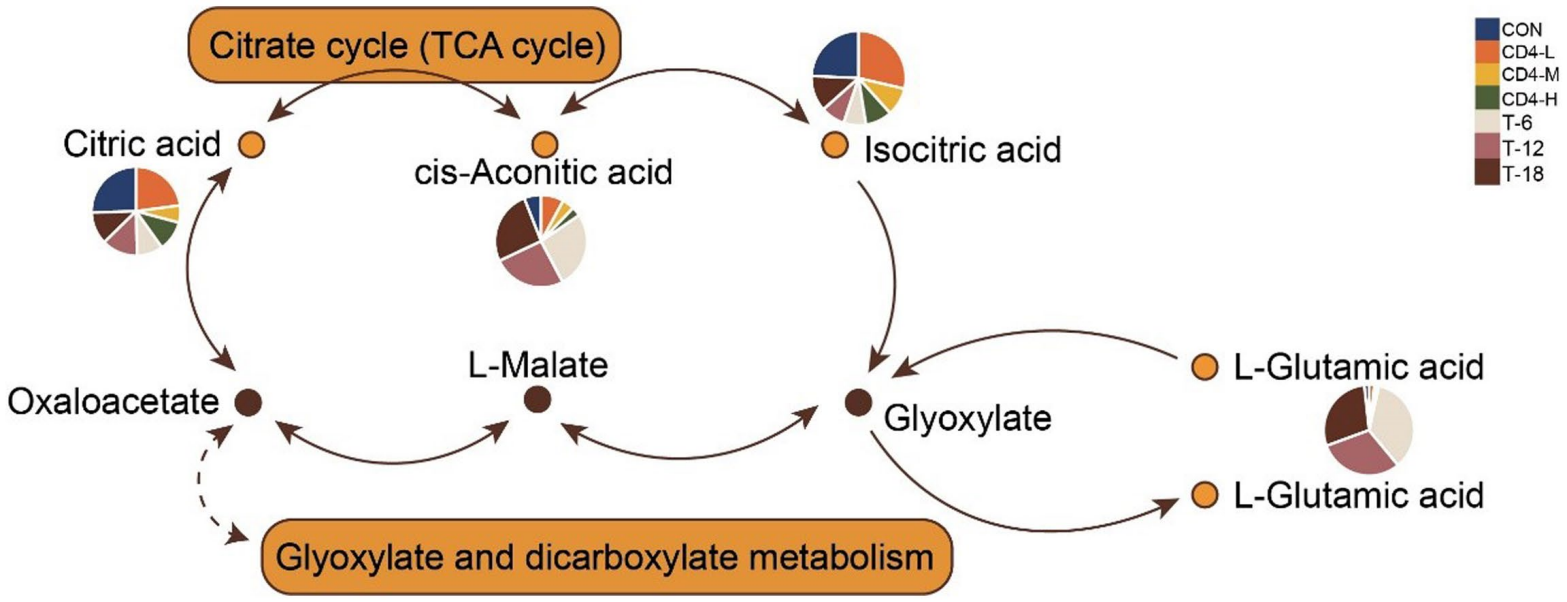
Combination of metabolic pathways in citrate cycle (TCA Cycle) and glyoxylate and dicarboxylate metabolism. The accumulation levels of involved metabolites are presented in pie charts within the pathway diagrams, with colors indicating group classification.

Differential and KEGG enrichment analyses of metabolites revealed that salivary metabolites might be associated with amino acid and energy metabolism after HIV infection, which was confirmed by subsequent analyses. The citrate cycle (TCA cycle) and glyoxylate and dicarboxylate metabolism were closely related to HIV. To further explore the accumulation pattern of specific metabolites detected within the two pathways, we visualized the levels of involved metabolites in pathway diagrams using pie charts. The pathways were interconnected through oxaloacetate, involving citric acid, cis-aconitic acid, and isocitric acid. Pathway diagrams indicated increased accumulation of is-aconitic acid and l-glutamic acid upon treatment, while citric acid and isocitric acid showed greater accumulation in the CD4-L group (Figure 5).

### 3.4 Autocorrelation analysis revealed key metabolite interactions in HIV

**Figure 6:**
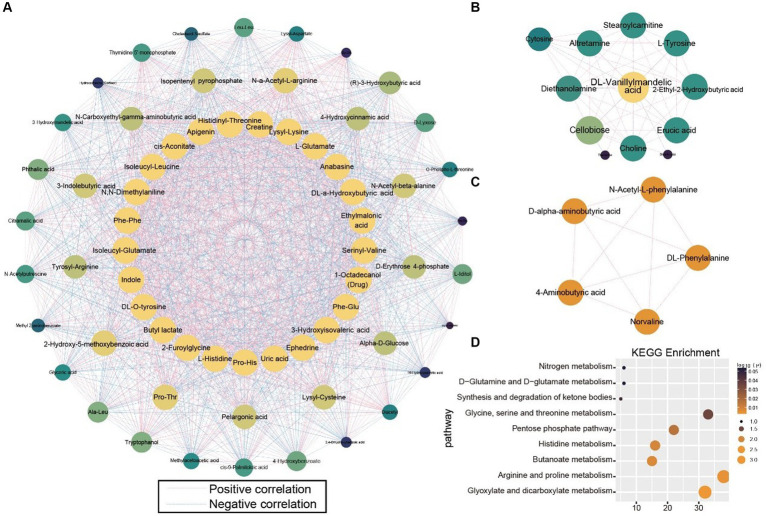
Correlation analysis of all metabolites. **(A–C)** Subnetworks with the highest scores in the metabolite correlation analysis. **(D)** KEGG enrichment analysis of metabolites in Subnetwork **(A)**.

To observe the antagonistic and synergistic interactions among metabolites, an autocorrelation analysis was performed based on the correlation coefficients of the 1,401 metabolites. Three networks with the highest MCODE scores were selected for further study (Figures 6A–C). Metabolites were ranked by degree value, with those in the inner circle of the network deemed more crucial, likely playing key roles. The network diagrams distinctly showed that the inner-circle metabolites were predominantly amino acids and exogenous substances. L-histidine, L-glutamate, indole, 3-hydroxyisovaleric acid, and serinyl-valine occupied central positions within the network, indicating their strong correlations with peripheral metabolites. N-α-acetyl-l-arginine and most of its connected metabolites exhibited positive correlations, suggesting a synergistic function. Conversely, isopentenyl pyrophosphate demonstrated negative correlations (Figure 6A). Dl-vanillylmandelic acid, positioned at the core of the network, was negatively correlated with erucic acid, 2-ethyl-2-hydroxybutyric acid, L-tyrosine, stearoylcarnitine, altretamine, diethanolamine, cellobiose, and choline, indicating an antagonistic role (Figure 6B). In the third subnetwork, five metabolites were mutually positively correlated (Figure 6C). To elucidate the functions of these metabolites in HIV infection more clearly, KEGG enrichment analysis was conducted on the metabolites from the first subnetwork. They were significantly enriched in pathways related to amino acid and energy metabolism, including glyoxylate and dicarboxylate metabolism, arginine and proline metabolism, butanoate metabolism, histidine metabolism, pentose phosphate pathway, glycine, serine, and threonine metabolism, synthesis and degradation of ketone bodies, d − glutamine and d − glutamate metabolism, and nitrogen metabolism (Figure 6D).

### 3.5 WGCNA identified distinct metabolite clusters in HIV infection

**Figure 7:**
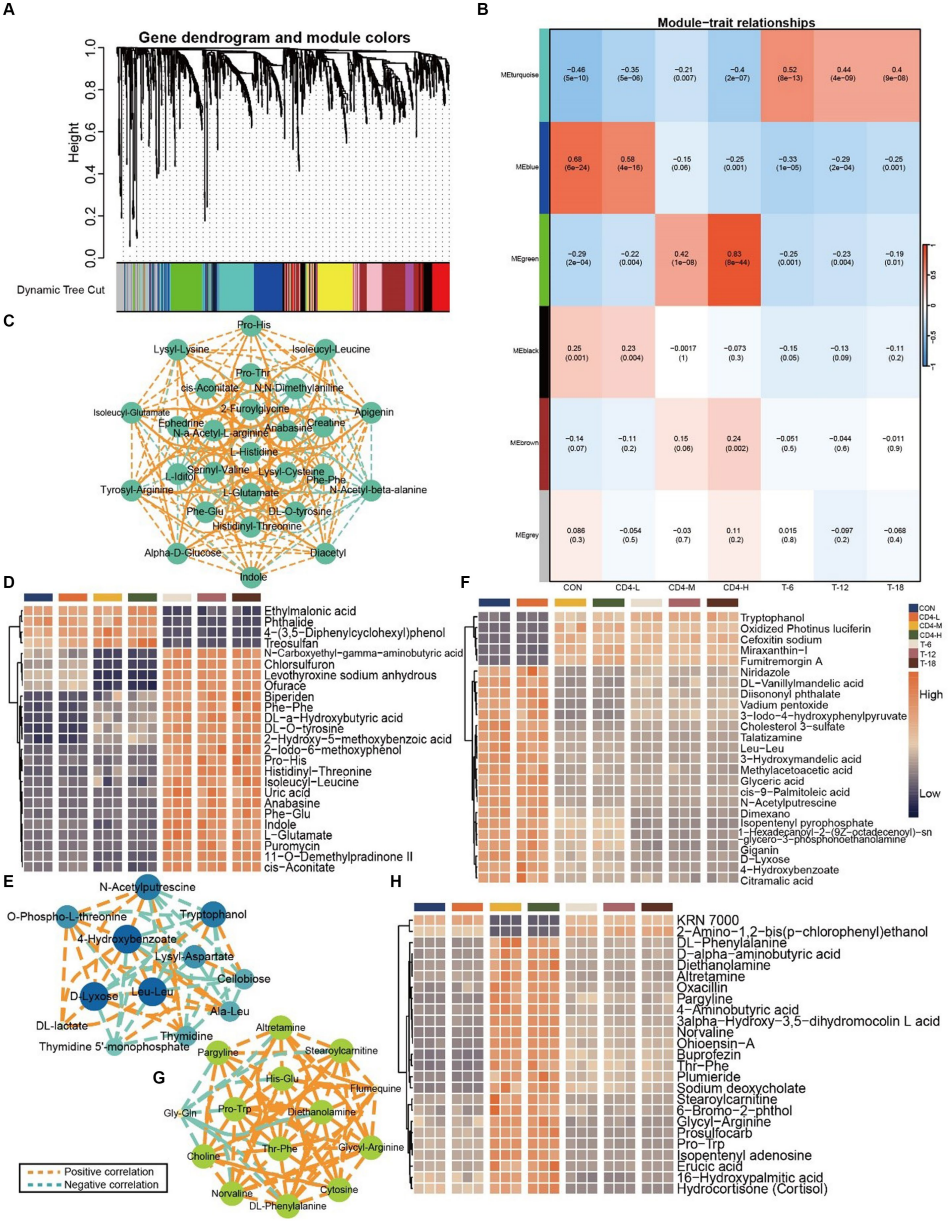
Metabolite rorrelation analysis across groups. **(A)** Dendrogram showing the clustering of the involved metabolites. **(B)** Heatmap of correlations between metabolites and the HIV and THIV groups by WGCNA, with correlation coefficients and corresponding *p*-values displayed in rectangles and brackets. **(C)** Correlation among metabolites in the turquoise module, with orange dashed lines indicating positive correlations and turquoise dashed lines indicating negative correlations. In the interaction network, each circle represents a metabolite, and each dashed line represents the interaction between correlated metabolites. Metabolite interactions are arranged in descending order of correlation level using Cytoscape; larger circles indicate greater relevance in the pathway. **(D)** Metabolite accumulation in the turquoise module, with the 25 most significant metabolites displayed by ANOVA (*p* < 0.05). **(E)** Correlation among metabolites in the blue module. **(F)** Metabolite accumulation in the blue module. **(G)** Correlation among metabolites in the green module. **(H)** Metabolite accumulation in the green module.

In the identification of pivotal metabolites, WGCNA was conducted. Within the WGCNA framework, all 1,401 metabolites were classified into distinct clusters, with the sample clustering dendrogram indicating well-defined sample grouping. Additionally, average linkage hierarchical clustering based on module distances resulted in the amalgamation of similar modules, culminating in a total of six distinct modules (Figures 7A,B). Subsequent analysis focused on metabolites from the turquoise (186), blue (170), and green (124) modules. Firstly, an assessment of inter-metabolite correlations within each module was carried out, followed by ANOVA of metabolite content, from which the 25 most significant metabolites were selected for visualization. In the correlation network diagram of the turquoise module, a predominance of positively correlated metabolites was observed, demonstrating similar levels of interconnectivity (Figure 7C). The accumulation of metabolites in the HIV group paralleled that of the CON group, while the THIV group exhibited an opposite trend. Following HAART, the level of ethylmalonic acid decreased, compared with untreated individuals. Metabolites such as histidinyl-threonine, isoleucyl-leucine, and L-glutamate showed increased levels after treatment (Figure 7D).

In the analysis of the blue module, 4-hydroxybenzoate, D-lyxose, and leu-leu exhibited the highest levels of interactivity. 4-Hydroxybenzoate was negatively correlated with tryptophanol and lysyl-aspartate, but positively with D-lyxose, O-phospho-L-threonine, and N-acetylputrescine (Figure 7E). The buildup of metabolites in the CON and CD4-L groups was consistent, while that in CD4-M, CD4-H, T-6, T-12, and T-18 groups was uniform. Oxidized photinus luciferin, cefoxitin sodium, and miraxanthin-I increased in treated groups, whereas glyceric acid, citramalic acid, and 3-iodo-4-hydroxyphenylpyruvate decreased in CD4-M, CD4-H, and THIV groups (Figure 7F). In the green module, the HIV group exhibited similar correlation strength for metabolites like pargyline, altretamine, stearoylcarnitine, diethanolamine, and DL-phenylalanine, except for gly-gln and flumequine. Gly-gln showed a negative correlation with other metabolites, whereas flumequine exhibited a positive correlation. The remaining metabolites demonstrated positive correlations (Figure 7G). The pattern in CD4-L was opposite to that in CD4-M and CD4-H, while the THIV group showed consistent metabolite accumulation. After treatment, KRN 7000 levels increased, whereas norvaline, glycyl-arginine, and other metabolites decreased (Figure 7H). These metabolites were identified as key players within their respective modules.

### 3.6 ROC and random forest analyses highlighted distinct metabolites between pre- and post-treatment stages

**Figure 8:**
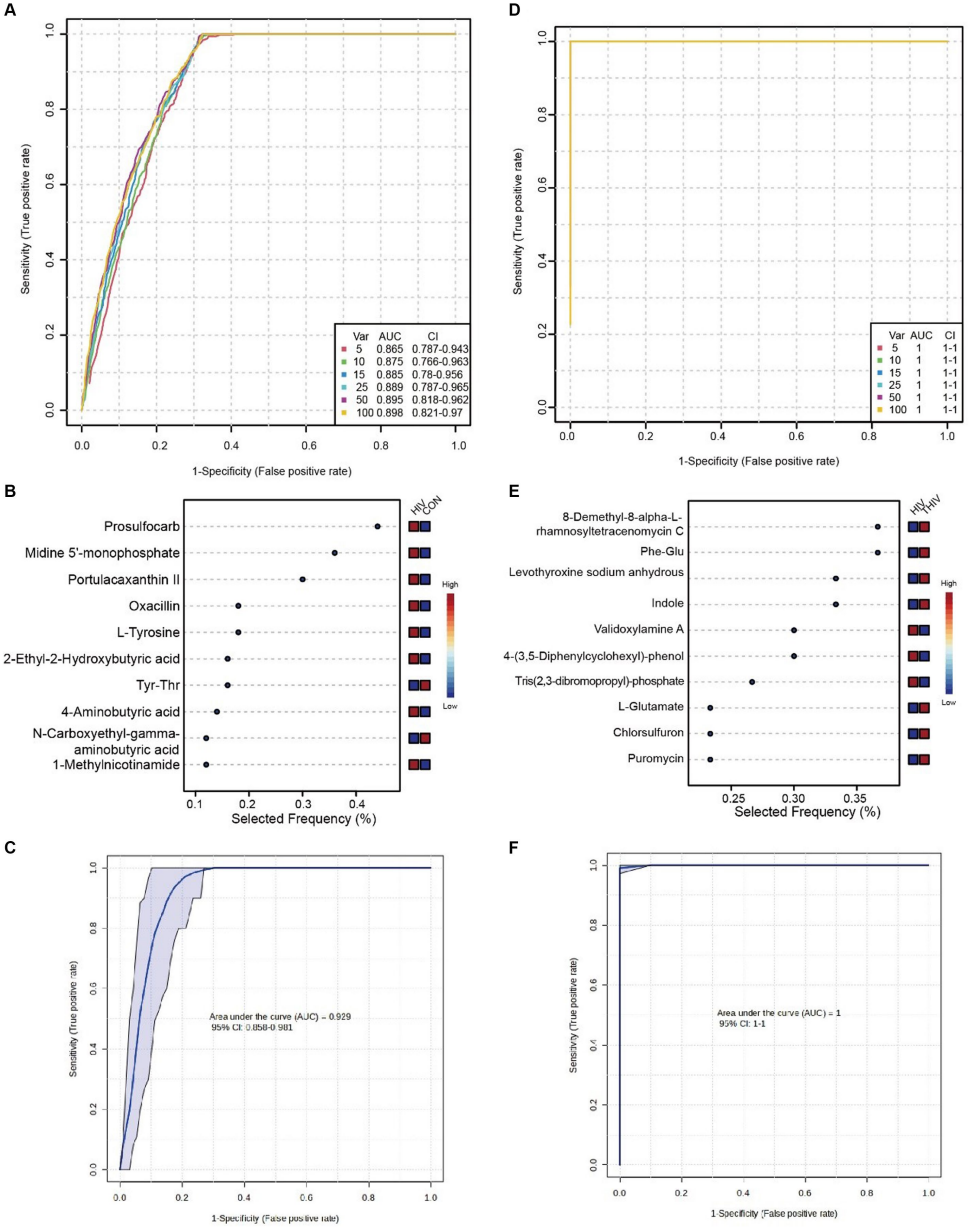
Biomarkers between HIV and THIV groups. **(A)** ROC curve based on cross-validation performance for metabolites between HIV and CON groups, with predicted class probabilities for each sample obtained using the best classifier based on AUC (mean of cross-validation). **(B)** Random forest analysis of metabolites between HIV and CON groups. **(C)** ROC curve for key metabolites validation between HIV and CON groups. **(D)** ROC curve based on cross-validation performance for metabolites between HIV and THIV groups. **(E)** Random forest analysis of metabolites between HIV and THIV groups. **(F)** ROC curve for key metabolites validation between HIV and THIV groups.

To discern key metabolites in pre- and post-treatment patients, ROC validation and random forest analysis were employed between CON and HIV groups, as well as between HIV and THIV groups. The multivariate classification model created by random forest showed an AUC of 0.885 when 15 metabolite factors were considered in the HIV group (Figure 8A). Metabolites such as L-tyrosine, oxacillin, portulacaxanthin II, midine 5′-monophosphate, and prosulfocarb had higher selection frequencies, effectively differentiating between the CON and HIV groups (Figure 8B). The ROC curve for these characteristic metabolites exhibited substantial sensitivity (AUC = 0.929) (Figure 8C). Additionally, in the comparison between HIV and THIV groups, the ROC curve achieved an AUC of 1 (Figure 8D), with validoxylamine A, indole, levothyroxine sodium anhydrous, Phe-Glu, and 8-Demethyl-8-alpha-L-rhamnosyltetracenomycin C being highly selected, thus effectively distinguishing between these groups (Figure 8E), as corroborated by the validation ROC curve (AUC = 1) (Figure 8F).

### 3.7 Oral metabolites in PLWH showed distinct correlations with herpes simplex virus type 1 (HSV1), bacteria, and fungi

**Figure 9:**
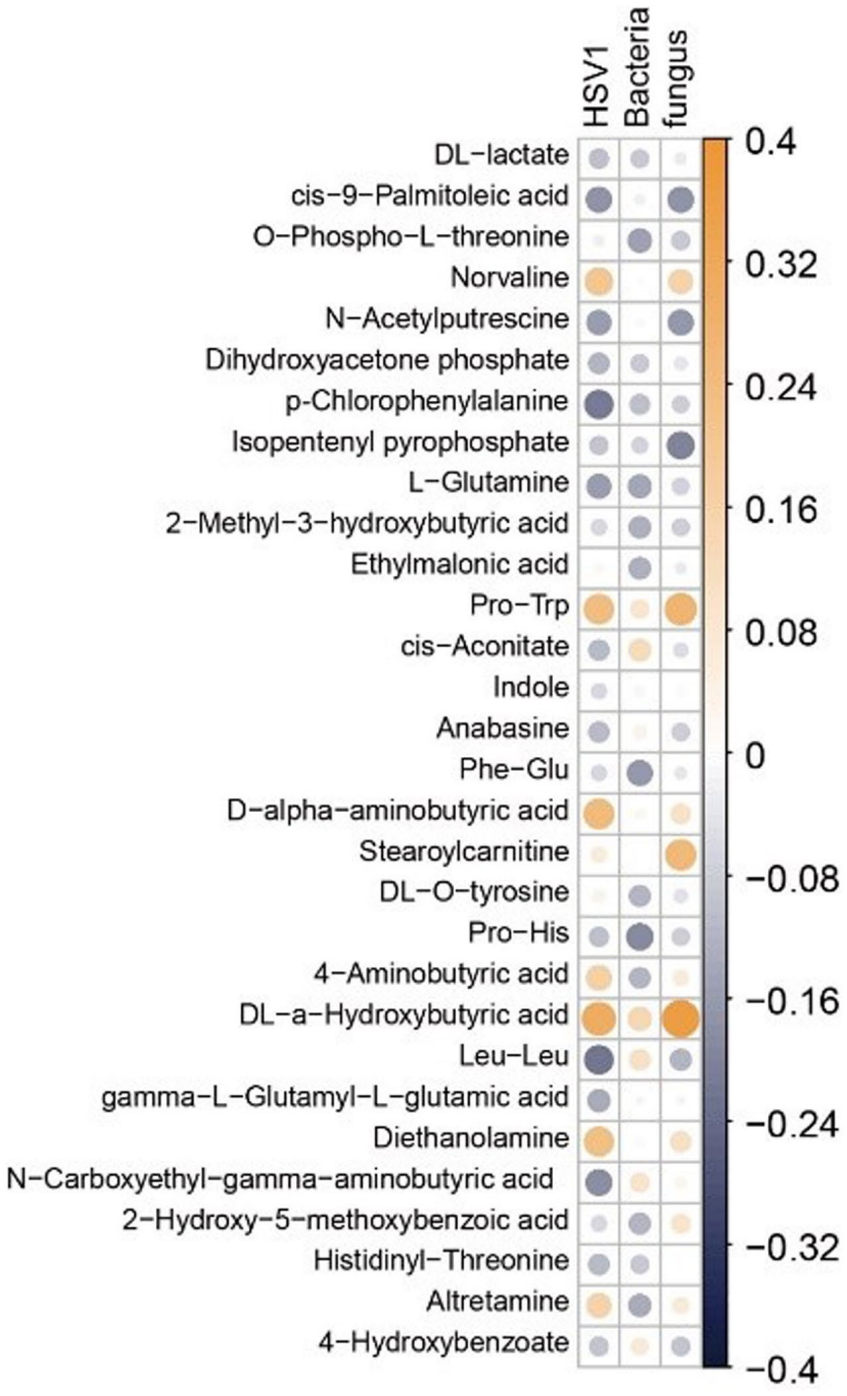
Heatmap of the correlation matrix between metabolites and microbes. Correlation coefficients are represented by a blue-white-yellow color scheme. Deep yellow indicates stronger positive correlations; deep blue indicates stronger negative correlations; white indicates no correlation. The top 30 metabolites are selected for display based on ANOVA.

In PLWH, the correlation between oral metabolites and HSV1, as well as oral-disease related bacteria and fungi was investigated due to the close association of HIV infection with various microbes. We performed the absolute quantification of HSV1, bacteria, and fungi in the saliva of PLWH. Subsequently, this quantification was correlated with the 30 most significant metabolites in the saliva of PLWH. The correlation matrix revealed a negative correlation between indole and HSV-1. Metabolites such as 2-Methyl-3-hydroxybutyric acid, Phe-Glu, O-Phospho-L-threonine, Pro-His, p-Chlorophenylalanine, gamma-L-Glutamyl-L-glutamic acid, isopentenyl pyrophosphate, L-Glutamine, and dihydroxyacetone phosphate showed negative correlations with HSV1, bacteria, and fungi. Conversely, stearoylcarnitine, Pro-Trp, diethanolamine, and DL-a-Hydroxybutyric acid exhibited positive correlations (Figure 9).

## 4 Discussion

### 4.1 Energy metabolism enhanced in saliva after HIV treatment

The application of untargeted metabolomics on saliva samples allows simultaneous detection of changes in both exogenous compounds and endogenous metabolites. This study has identified that the citrate cycle (TCA cycle) and glyoxylate and dicarboxylate metabolism play pivotal roles upon HIV infection. Acetyl coa condenses with oxaloacetic acid under the action of citrate synthetase to form citric acid, which is subsequently converted to isocitric acid by aconitase. Although these two pathways share metabolic components and are involved in energy metabolism, they exhibit distinct differences. The glyoxylate cycle in glyoxylate and dicarboxylate metabolism represents the conversion of fat to sugar, commonly considered as an ancillary route to the TCA cycle, which is central to cellular metabolism. Conversely, the TCA cycle occurs in mitochondria and is closely associated with oxidative decarboxylation of sugars (21, 22). The TCA cycle, being central to carbon and energy metabolism and a primary source of cellular energy, has been found to have its intermediates in serum acting as biomarkers for various potential pathological conditions (23, 24). Despite enrichment of the TCA cycle discovered in saliva exposome-wide association studies (EWAS) (25), research focusing on the salivary metabolites of PLWH remains scarce. Studies have shown that the TCA cycle is the most significantly altered metabolic pathway in the cerebrospinal fluid of people living with PLWH, affecting other amino acid and lipid metabolisms (26).

Citric acid, reversibly converted to cis-aconitic acid and isocitric acid under the action of aconitase, undergoes changes in the cerebrospinal fluid metabolism of PLWH. Accumulation of citric acid could potentially lead to cognitive deterioration in patients (26, 27), suggesting its harmful role in the progression of HIV. In the CD4-L group (CD4 count <200/mm3), the accumulation of citric acid was significantly higher than in the CD4-M, CD4-H, and other post-treatment groups. This result implied that worsening conditions in PLWH might increase the accumulation of citric acid in saliva, leading to adverse disease progression. *In vitro* inhibition experiments have shown that using succinic acid or cis-aconitic acid for acylation of lysine residues in proteins results in derivatives that exhibit potent antiviral activity against HIV-1 and/or HIV-231. Our analysis revealed that post-treatment patients had significantly higher levels of cis-aconitic acid compared with untreated individuals, suggesting that increased cis-aconitic acid levels may enhance antiviral activity in the body. Hence, the level of cis-aconitic acid in saliva could potentially serve as an indicator of recovery in PLWH.

Glutamic acid has been extensively studied in the context of endogenous anticancer agents and anticancer drug conjugates, demonstrating its effectiveness in these areas (28). Furthermore, the synthesis of L-glutamic acid amide has shown activity against Ehrlich ascites carcinoma (29). The HIV-1 Gag p6 protein regulates the final steps of new viral particle detachment from the cell membrane through the action of its two late domains. Glutamic acid within P6 contributes to late-stage viral replication and may facilitate interactions between Gag and the lipid membrane (30). Additionally, L-glutamic acid is converted to L-glutamine by L-glutamine synthetase, and studies have indicated alterations in L-glutamine metabolism in CD4+ T cells infected with HIV-1 (31). In our study, the treatment group exhibited higher levels of L-glutamic acid in saliva compared with other groups. As L-glutamic acid is an upstream metabolite of L-glutamine, we hypothesize that HAART promotes the synthesis of L-glutamic acid. Pathway analysis revealed increased levels of metabolites related to glyoxylate and dicarboxylate metabolism and the tricarboxylic acid (TCA) cycle in saliva after treatment, suggesting enhanced energy metabolism. Based on these findings, we propose that treatment of PLWH may lead to an increase in energy metabolism in saliva.

### 4.2 Metabolites in the saliva of HIV individuals were primarily enriched in energy metabolism pathways, and their levels changed with the reduction in CD4 cells

Upon pathway analysis, it was observed that metabolite pathways in PLWH saliva were predominantly related to energy metabolism, distinguishing CD4-L from CD4-M and CD4-H groups. Results indicated that when CD4 counts was between 200–500 cells/mm3, metabolite levels underwent alterations compared with the control group. However, when CD4 counts dropped below 200 cells/mm3, metabolite levels changed again, gradually returning to normal levels. In some studies, HIV has been linked to innate and adaptive immune activation. Furthermore, immunological activation associated with mycobacterial infections or autoimmune diseases has been found to significantly alter the metabolic state of the immune system, disrupting metabolism and affecting the host response to pathogens, leading to metabolic disturbances (32, 33).

Given that HIV infection is a persistent, long-term, and challenging condition to treat, it is postulated that as the disease progresses, saliva metabolite levels gradually stabilize. Additionally, since the control group comprises HIV-negative individuals, the changes in saliva metabolite levels are less pronounced. These factors may contribute to the observed phenomenon, although further research is needed to determine the specific reasons. Research has shown an increase in the relative abundance of N-acetylputrescine following HIV infection, which is consistent with our findings (34). The anti-inflammatory properties of adenosine can regulate chronic inflammation and immune activation induced by HIV. As HIV progresses, CD4-L patients exhibit an increase in adenosine levels in saliva. Moreover, research suggests that HIV infection directly or indirectly induces dopamine dysfunction (35), a finding also supported by our results. Some studies have reported significantly lower levels of linoleic acid and argininosuccinic acid in individuals with HIV-1 (36, 37), and an increase in brain choline compounds before the onset of AIDS dementia in PLWH (38). Furthermore, palmitic acid has been studied for its ability to inhibit HIV-1 infection by blocking effective attachment between gp120 and CD4 (39). Our analysis revealed a significant decrease in the levels of sphinganine, linoleic acid, and argininosuccinic acid in the CD4-L group, suggesting that the metabolic trends of these metabolites in saliva align with those in blood. Although choline levels were lower in CD4-L, they were higher in CD4-M and CD4-H, indicating that choline metabolism in saliva partially mirrored the metabolic trends in blood. Additionally, cluster 7 in the trend analysis clearly showed the changes in these metabolites, with their levels gradually increasing from early to late-stage HIV infection. Based on this analysis, it can be inferred that these metabolites may serve as potential biomarkers for HIV infection in saliva.

AIDS is a disease characterized by a prolonged course and intricate mechanisms, with its severity closely tied to immune levels. Plasma markers have been pivotal in tracking changes (40), yet hematological examinations pose risks, including transient pain, severe bleeding, and even disease transmission through medical exposure in PLWH. Therefore, identifying non-invasive biomarkers to delineate the physiological and pathological processes of HIV infection is of great significance. Saliva markers emerge as promising candidates, offering convenient sampling, safety, and negligible risk of disease transmission to others.

### 4.3 A substantial presence of exogenous metabolites was detected in the saliva of PLWH with the introduction of HAART, involving multiple amino acid metabolic pathways

Following HAART, significant changes occurred in the saliva metabolites of PLWH, with most prominently affected metabolites being exogenous, such as chlorsulfuron, oxidized photinus luciferin, cefoxitin sodium, and levothyroxine sodium anhydrous. These metabolites showed increased levels by treatment, indicating the detection of a considerable amount of exogenous metabolites in saliva. Additionally, our analysis identified enriched metabolic pathways besides the TCA cycle and glyoxylate and dicarboxylate metabolism. These included butanoate metabolism, lysine degradation, and arginine and proline metabolism. Creatine, associated with arginine and proline metabolism, demonstrated an elevation in saliva content after treatment. Research has indicated that creatine has the potential to inhibit mitochondrial depolarization induced by human immunodeficiency virus-1 transcription activator (HIV-1 TAT) and the opening of mitochondrial permeability transition pores triggered by HIV-1 TAT. These findings suggest a role in the treatment of HIV-1-associated neurocognitive disorders (41). Plasma N-acetylputrescine has also been utilized as a potential biomarker for assessing lung cancer treatment (42). Based on our research, these metabolites are suggested as potential biomarkers detectable in the saliva of PLWH, but their specific roles require further validation. Trend analysis revealed three distinct patterns of change in metabolite levels, with functional relevance mainly observed in energy metabolism (fatty acid biosynthesis, starch and sucrose metabolism) and overall body metabolism.

### 4.4 Indoles and L-glutamine may exhibit antagonistic effects against HSV-1, bacteria, and fungi

PLWH are susceptible to bacterial infections, a factor that further complicates their condition (43). Additionally, certain fungi can exert a detrimental impact on immunocompromised patients, frequently leading to invasive diseases (44). Moreover, HSV-1 infection increases the incidence of HIV-related illnesses (45). Therefore, monitoring changes in oral microbiota can aid in understanding and controlling HIV progression. Correlation analysis revealed a relationship between microorganisms and metabolites in saliva in the context of HIV infection. Indole, a degradation product of tryptophan, serves as a signaling molecule in many bacteria (46). Indoles exhibit significant biological activities in antioxidative, anti-inflammatory, anti-fungal, and antibacterial effects (47, 48). In our work, indole showed a negative correlation with HSV1, suggesting its inhibitory effect on HSV1 development. Glutamine has been identified for its role in critical illnesses, cancer, and heart injury (49), and L-glutamine has been found to assist in alleviating nelfinavir-associated diarrhea in PLWH (50). L-glutamine exhibits a negative correlation with HSV-1, bacteria, and fungi, further supporting its inhibitory effect on these microbes.

## Data availability statement

The raw data supporting the conclusions of this article will be made available by the authors, without undue reservation.

## Ethics statement

The studies involving humans were approved by Medical Ethics Committee of Yan’an Hospital in Kunming, Yunnan Province. The studies were conducted in accordance with the local legislation and institutional requirements. The participants provided their written informed consent to participate in this study.

## Author contributions

FD: Data curation, Formal analysis, Methodology, Visualization, Writing – original draft, Writing – review & editing. RL: Data curation, Formal analysis, Writing – original draft, Writing – review & editing. RH: Methodology, Project administration, Resources, Writing – review & editing. KL: Methodology, Writing – review & editing. JL: Methodology, Resources, Writing – review & editing. YX: Methodology, Writing – review & editing. KD: Conceptualization, Funding acquisition, Supervision, Writing – review & editing. CL: Conceptualization, Funding acquisition, Project administration, Writing – review & editing.

## Footnotes

^1^ https://www.kegg.jp/kegg/pathway.html

^2^ www.arb-silva.de
